# Evolving landscape of economic evaluations of HIV pre‐exposure prophylaxis and pre‐exposure prophylaxis implementation strategies: a systematic review

**DOI:** 10.1002/jia2.70058

**Published:** 2025-11-23

**Authors:** Min Xi, Darrell H. S. Tan, Stefan D. Baral, Howsikan Kugathasan, Lisa Masucci, Becky Skidmore, Derek R. MacFadden, Kednapa Thavorn, Sharmistha Mishra

**Affiliations:** ^1^ Ottawa Hospital Research Institute The Ottawa Hospital Ottawa Ontario Canada; ^2^ Dalla Lana School of Public Health University of Toronto Toronto Ontario Canada; ^3^ Toronto General Hospital Research Institute University Health Network Toronto Ontario Canada; ^4^ Institute of Health Policy Management, and Evaluation University of Toronto Toronto Ontario Canada; ^5^ MAP Centre for Urban Health Solutions Li Ka Shing Knowledge Institute St Michael's Hospital Unity Health Toronto Toronto Ontario Canada; ^6^ Division of Infectious Diseases St Michael's Hospital Unity Health Toronto Toronto Ontario Canada; ^7^ Department of Medicine Faculty of Medicine University of Toronto Toronto Ontario Canada; ^8^ Institute of Medical Science University of Toronto Toronto Ontario Canada; ^9^ Department of Epidemiology Johns Hopkins University Baltimore Maryland USA; ^10^ Toronto Health Economics and Technology Assessment Collaborative Toronto General Hospital Research Institute University Health Network Toronto Ontario Canada; ^11^ Skidmore Research & Information Consulting Inc. Ottawa Ontario Canada; ^12^ ICES Toronto Ontario Canada; ^13^ School of Epidemiology and Public Health University of Ottawa Ottawa Ontario Canada

**Keywords:** HIV, pre‐exposure prophylaxis, cost‐benefit analysis, cost‐effectiveness analysis, mathematical model, HIV epidemic

## Abstract

**Introduction:**

Economic evaluations of HIV pre‐exposure prophylaxis (PrEP) and associated implementation strategies guide evidence‐based policies, programmes and resource allocation. Since 2015, there has been an evolution in PrEP modalities, implementation strategies and prioritization of key populations with unmet HIV prevention needs, alongside the scale‐up of other HIV prevention interventions. Our systematic review describes the evolving landscape of economic evaluations of PrEP to help identify evidence gaps relevant to the current HIV epidemic and response (PROSPERO: CRD42016038440).

**Methods:**

We searched five databases, without language restrictions, for peer‐reviewed economic evaluations from inception to 21 August 2025. We describe the evolution of study characteristics over time, including the perspective of analysis, region, population, PrEP modality/implementation strategy and comparators.

**Results:**

Of 5400 studies identified, 128 met inclusion criteria, of which 94 examined HIV epidemics in 2015 or later and 17 adopted a societal perspective. HIV epidemics studied primarily spanned countries in sub‐Saharan Africa (*N* = 51) and in North America (*N* = 34). Modelled populations for receipt of PrEP primarily comprised: gay, bisexual and other men who have sex with men (*N* = 73), female sex workers (*N* = 26), serodifferent partnerships (*N* = 17) and persons who inject drugs (*N* = 12). Most evaluated oral, daily PrEP (*N* = 76), followed by long‐acting injectable PrEP (*N* = 17), on‐demand PrEP (*N* = 16) and others (e.g. vaginal ring, topical gel; *N* = 7). Twelve studies compared different PrEP modalities with each other. Five studies evaluated different implementation strategies to increase PrEP uptake, adherence and persistence. Of the 123 studies that compared PrEP to a combination of other HIV prevention interventions, only 31 scaled up at least part of the comparator over time.

**Discussion:**

To support decision‐making, future economic evaluations should consider costs and benefits beyond the health system (society) and consider comparators that better reflect the current HIV response across regions and populations. The increasing availability of novel PrEP modalities allows future studies to evaluate a mix of PrEP modalities and person‐centred implementation strategies.

**Conclusions:**

The growing number of PrEP economic evaluations have not kept pace with emerging PrEP modalities or the current HIV epidemic/response, resulting in challenges in making evidence‐based policies, programmes and resource allocation.

## 1 INTRODUCTION

The last decade has seen an expansion in the adoption and rollout of pre‐exposure prophylaxis (PrEP) for HIV prevention. Daily oral PrEP, consisting of emtricitabine and either tenofovir disoproxil fumarate or tenofovir alafenamide, was first authorized for use in the United States in 2012 [1]. By 2015, PrEP became a key component of combination HIV prevention globally when the World Health Organization recommended oral PrEP for individuals at “substantial risk” of acquiring HIV (annual HIV risk greater than 3%) [2]. The World Health Organization later endorsed “on‐demand” oral PrEP in 2019 [3]. In 2020, the European Medicines Agency endorsed topical PrEP modalities [4]. More recently, long‐acting injectable PrEP (cabotegravir and lenacapavir) was approved for use in the United States in 2021 [5] and 2025 [6], respectively. In addition to new modalities, the last decade has also seen the adoption of various PrEP implementation strategies (e.g. mobile phone applications [7] or online modules [8] with information on PrEP or peer support for PrEP initiation and persistence). By 2021, 144 countries recommended oral PrEP within their national HIV prevention guidelines, with 14 more planning to adopt PrEP into their guidelines within the next 2 years [9].

PrEP implementation strategies have been a central focus for HIV programmes in the last decade [10, 11] because, despite the inclusion of PrEP in national guidelines, coverage remains low in many countries [12]. PrEP use in 2020 was at 8% of the 2025 global target set by UNAIDS [13]. While individual‐level efficacy in preventing HIV acquisition is high (upwards of 99% [14]), real‐world individual‐level or partnership‐level effectiveness has been lower [15, 16, 17, 18], partly due to challenges with adherence and persistence [19, 20]. Although mathematical modelling studies suggest large potential for reducing sexual transmission of HIV at the population‐level, such outcomes depend on PrEP reaching individuals with the most unmet prevention needs who are part of sexual networks with high rates of HIV transmission [21, 22, 23].

Economic evaluations of PrEP and PrEP implementation strategies are valuable for informing policy decisions, optimizing resource allocation and guiding the adoption of different implementation strategies [24]. To date, there have been 12 reviews of economic evaluations of PrEP [25, 26, 27, 28, 29, 30, 31, 32, 33, 34, 35, 36], but they were largely restricted with respect to geography and/or populations. Two reviews synthesized evidence from a single country (United States) [25, 26], three were restricted to a subset of countries (high‐income, early PrEP adopting countries) [34, 36] or to one region (sub‐Saharan Africa) [35] and two focused on specific populations (gay, bisexual and other men who have sex with men; persons who inject drugs) [25, 26]. These earlier reviews were also restricted to economic evaluations conducted prior to the recent expansions in PrEP rollout and expansions in PrEP modalities and implementation strategies [25, 26, 27, 28, 29, 30, 31, 32].

The evolution of PrEP rollout over the last decade signals a need to characterize how economic evaluations may have evolved (e.g. the examination of different PrEP modalities and implementation strategies) to keep pace with implementation‐relevant questions for decision‐makers and communities most affected by HIV [11]. The most recent systematic review, which included economic evaluations conducted between 2010 and 2020, was not designed to describe the evolution of comparators used in PrEP economic evaluations over time and whether these comparators reflected the evolving HIV epidemic response [33] (e.g. implementation and scale‐up of concurrent HIV prevention tools, strategies and guidelines [37, 38, 39]). For example, comparison of PrEP to steady levels of other modes of primary and secondary HIV prevention at a population level may be less applicable for decision‐making in recent years, especially given the acceleration in sustained viral suppression as secondary HIV prevention [40]. The scale‐up of combination HIV prevention interventions has led to large reductions in HIV incidence globally, including in countries with some of the highest burden of HIV, and incidence reductions in populations at highest risk of HIV acquisition (including gay, bisexual and other men who have sex with men, cisgender female sex workers and other people engaged in sex work, transgender women, persons who inject drugs) [41]. Therefore, the questions being asked by decision‐makers involve comparing and choosing between different PrEP modalities and implementation strategies [42]. In doing so, decision‐makers seek strategies to improve each step of the PrEP cascade [42] (including persistence [20]) and determine where and among whom new HIV acquisitions continue to be acquired despite local efforts in scaling up combination HIV prevention [41].

In this study, we sought to: (1) describe the evolving landscape of economic evaluations of PrEP and its implementation strategies over time and across epidemic contexts (population focus, country/region) and (2) highlight gaps in the literature. Our goal was to provide guidance for future economic evaluations (e.g. which PrEP interventions/implementation strategies to evaluate and which comparators, HIV epidemic context, and perspective should be used during analyses) to better support decision‐making in the evolving landscape of PrEP implementation.

## 2 METHODS

Our systematic review followed the Preferred Reporting Items for Systematic Reviews and Meta‐Analyses (PRISMA) guidelines (online Appendix A) [43]. Our full review protocol was peer‐reviewed and published [44] and registered in PROSPERO (registration number CRD42016038440). Our full review protocol objectives included: (1) estimating the incremental cost per health outcome of PrEP or PrEP implementation strategies compared to placebo, status quo or other HIV prevention strategies; (2) assessing the variability in and quality of economic evaluations of PrEP or PrEP implementation strategies; and (3) identifying potential sources of heterogeneity in the cost‐effectiveness of PrEP or PrEP implementation strategies. In this first paper, we described the trends and characteristics of PrEP economic evaluations over time and across epidemic contexts, with a focus on relevance to evolving decision‐making needs in the current stage of the global HIV epidemic—specifically, regarding PrEP modalities, implementation strategies and comparators [41]. Research Ethics Board review was not required as all data used in this review were derived from previously published studies. No human participant data were used, so consent was not required.

### 2.1 Search strategy and inclusion/exclusion criteria

An information specialist (BS) developed the strategy in consultation with the review team. The strategies were executed in Ovid MEDLINE® ALL, Embase Classic+Embase (Ovid) and the NHS Economic Evaluation Database (Wiley). An initial search was conducted to identify articles published from inception to 30 August 2019, updated to 23 December 2021, updated to 21 December 2024 and updated again to 21 August 2025. The NHS Economic Evaluation Database was not included in the updated search as it was no longer being updated and was removed from the Cochrane Library. The search included a combination of controlled vocabulary (e.g. “HIV Infections/pc [Prevention & Control], ” “Pre‐Exposure Prophylaxis, ” “Models, Economic”) and free‐text terms (e.g. “HIV prevention, ” “PrEP, ” “economic evaluation”). There were no language or date restrictions but, where possible, animal‐only records and conference abstracts were removed from the results. The full search strategy is available in online Appendix B. We (MX) also conducted a bibliographic hand search of relevant reviews to identify studies that may have been missed by the electronic database searches.

We included full economic evaluations (i.e. cost‐minimization analyses, cost‐benefit analyses, cost‐effectiveness analyses or cost‐utility analyses) that compared both costs and outcomes of PrEP or of a PrEP implementation strategy [45]. Studies could compare PrEP as an intervention to placebo, to other HIV prevention interventions (e.g. antiretroviral therapy [ART], condoms, HIV testing, HIV counselling, male circumcision), or to other PrEP modalities; and/or compare different PrEP implementation strategies to each other. We excluded editorials, conference abstracts, preprints and review articles. We excluded studies evaluating emtricitabine and either tenofovir disoproxil fumarate or tenofovir alafenamide for post‐exposure prophylaxis rather than PrEP. We excluded economic evaluations focused solely on the prevention of parent‐to‐child transmission of HIV.

Following duplicate removal, titles and abstracts were screened independently by two reviewers (two of either MX, KT or SM) for inclusion/exclusion using Covidence (Veritas Health Innovation, Melbourne, Australia). For included titles/abstracts and in cases where the title and abstract were deemed insufficient for an exclusion decision, two reviewers (two of either MX, HK, KT or SM) independently conducted full‐text screening against the inclusion/exclusion criteria. Discrepancies during title and abstract, and full‐text screening were resolved by consensus.

### 2.2 Data extraction

Data from the included publications were extracted by one reviewer (MX, LM, HK or NM) and verified by a second reviewer (MX, LM, HK, NM or OD). We extracted data on study characteristics, including the type of economic evaluation conducted, whether the economic evaluation involved a knowledge user and whether the economic evaluation was nested within a clinical trial or real‐world implementation programme. For study purposes, we categorized the remaining extracted variables as follows: (1) epidemic context; (2) perspective of analyses; (3) intervention; and (4) comparator (online Appendix C).

Epidemic context pertained to the underlying epidemic and population under study, with variables including country and population who received PrEP and populations included in the outcome measures. Variables related to the underlying epidemic included: country and World Bank region [46], publication year and model time‐horizon, HIV epidemic period modelled and calendar time‐period of PrEP use. Populations included population focus, such as gay, bisexual and other men who have sex with men, persons who inject drugs, female sex workers, male sex workers, HIV‐serodifferent partners, adolescent girls and young women; and the wider population (e.g. total adult population).

For the perspective of analyses, we extracted data on whether evaluations were conducted using a health system, societal perspective and/or other perspective. Intervention variables included: concurrent interventions (e.g. concomitant ART, condoms, etc.); PrEP modality (oral [daily or on‐demand], continuous, long‐acting injectable, vaginal ring, topical gel); PrEP implementation strategies (e.g. PrEP provider strategy, strategies to increase PrEP coverage and/or to improve adherence to PrEP).

For comparator‐related variables, we extracted the comparator type (e.g. ART alone; ART with other HIV prevention interventions excluding PrEP; other prevention interventions without ART). If PrEP modality or implementation strategies were evaluated, then comparators could also include: alternate PrEP modalities with or without ART and/or other prevention interventions; alternate PrEP implementation strategies with or without ART and/or other prevention interventions. If comparators did not fall into the above categories, we classified them as “other” and provided details in the extraction form. Finally, we extracted data on whether any of the interventions included in the comparator were scaled up during the period of comparative analyses (e.g. increased coverage of ART) and the trend in the modelled HIV incidence in the comparator scenario.

### 2.3 Data synthesis

We conducted a narrative synthesis of economic evaluations and summarized the results in tabular and graphical formats (R version 4.2.0 [47]). We described the following characteristics of the included studies: epidemic context, perspective of analysis, PrEP modality or implementation strategy and comparator(s). We examined whether comparators used in analyses included the use and scale‐up of various modes of primary and secondary population‐level HIV prevention interventions [37, 38, 40]. Specifically, we quantified the number of studies that included ART or other HIV prevention interventions in the comparator and whether models accounted for the increased uptake/coverage of at least one of these HIV prevention interventions over time. For studies that compared one PrEP modality or implementation strategy to another, we described the PrEP modality, HIV prevention interventions and/or PrEP implementation strategies included in the comparator. Results were stratified by year of publication to show the evolution of the landscape of PrEP and PrEP implementation economic evaluation studies over time. We also quantified the number of studies published in 2015 or later, following the World Health Organization endorsement of PrEP [2].

## 3 RESULTS

### 3.1 Study selection

**Figure 1:**
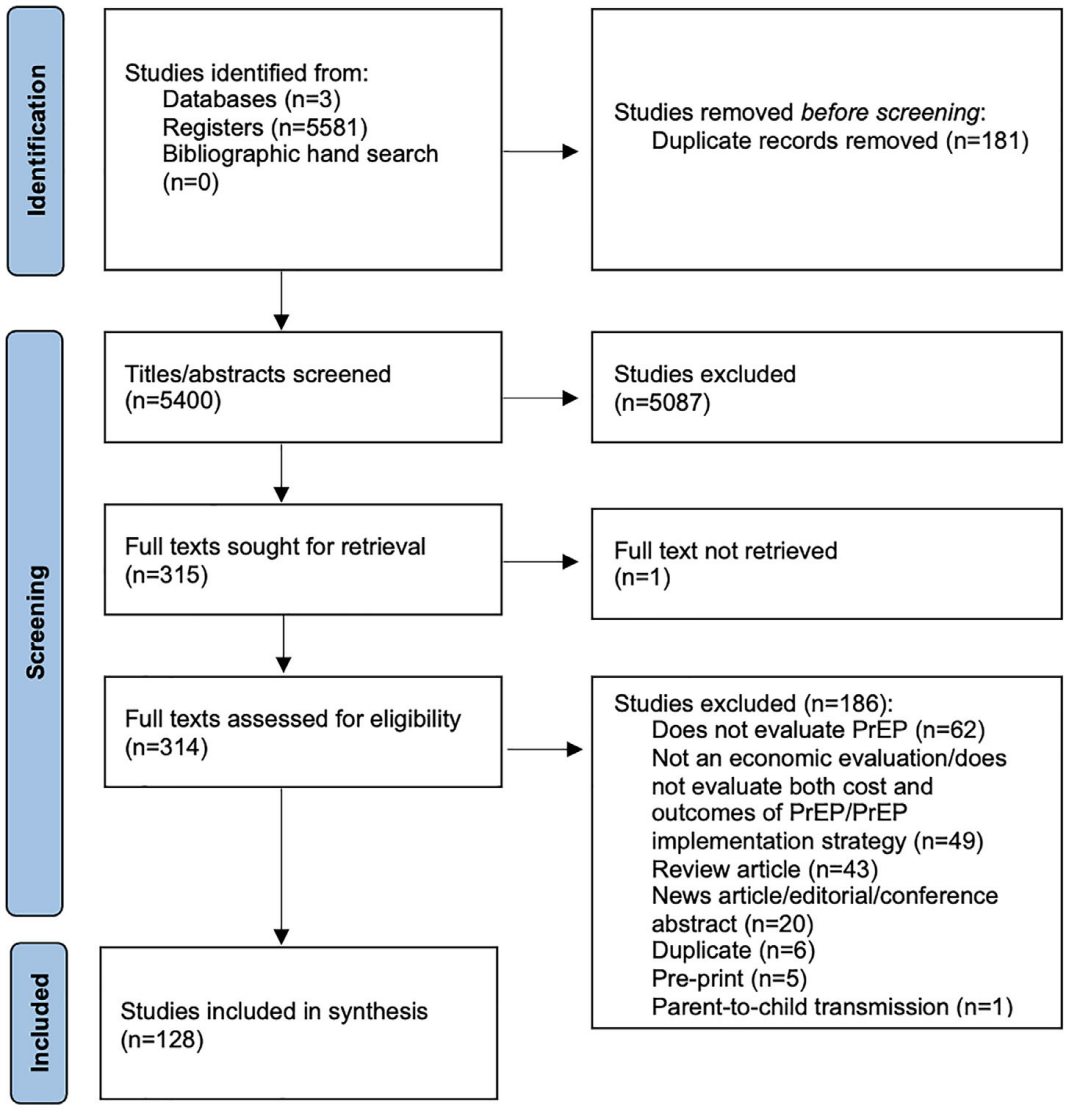
PRISMA flow diagram of study selection. Abbreviation: *n*, number of studies.

Figure 1 describes the study inclusion process. Our search identified 5400 unique records. Following title and abstract screening, 314 studies were retrieved for full‐text screening. One hundred and twenty‐eight studies met the inclusion criteria and were included in our review. No additional studies were identified through a bibliographic hand search.

### 3.2 Key study characteristics

Of the 128 included studies, 92 (72%) were cost‐utility analyses (i.e. assessed cost per quality adjusted or disability‐adjusted life year gained) [48, 49, 50, 51, 52, 53, 54, 55, 56, 57, 58, 59, 60, 61, 62, 63, 64, 65, 66, 67, 68, 69, 70, 71, 72, 73, 74, 75, 76, 77, 78, 79, 80, 81, 82, 83, 84, 85, 86, 87, 88, 89, 90, 91, 92, 93, 94, 95, 96, 97, 98, 99, 100, 101, 102, 103, 104, 105, 106, 107, 108, 109, 110, 111, 112, 113, 114, 115, 116, 117, 118, 119, 120, 121, 122, 123, 124, 125, 126, 127, 128, 129, 130, 131, 132, 133, 134, 135, 136, 137, 138, 139], 42 (33%) were cost‐effectiveness analyses (e.g. assessed cost per HIV infection averted) [71, 74, 83, 102, 104, 105, 109, 117, 119, 130, 137, 138, 140, 141, 142, 143, 144, 145, 146, 147, 148, 149, 150, 151, 152, 153, 154, 155, 156, 157, 158, 159, 160, 161, 162, 163, 164, 165, 166, 167, 168, 169], two (2%) were cost‐benefit analyses [137, 152] and nine (7%) were budget impact studies [7, 88, 116, 134, 170, 171, 172, 173, 174]. Of the included studies, only 13 (10%) were conducted alongside a clinical trial or real‐world implementation programme [7, 65, 93, 97, 105, 108, 122, 125, 128, 136, 137, 145, 168] and nine engaged knowledge users such as people living with HIV and policymakers [53, 54, 76, 77, 111, 132, 146, 157, 159].

### 3.3 Timeline in number of economic evaluations by key events

We identified an increasing trend in the number of economic evaluations that assessed the cost‐effectiveness of PrEP in recent years. Six studies (5%) [58, 84, 102, 149, 158, 163] were published between 2008 and 2011 prior to US FDA approval of PrEP [1], 15 studies (12%) [49, 50, 55, 64, 67, 73, 78, 79, 90, 92, 94, 98, 142, 153, 161] were published between 2012 and 2014 following US FDA approval of PrEP [1], 20 studies (16%) [59, 66, 68, 74, 75, 76, 80, 83, 85, 89, 105, 140, 143, 144, 147, 155, 160, 162, 170, 171] were published between 2015 and 2016 following WHO endorsement of PrEP [2] and 87 studies (68%) [7, 48, 51, 52, 53, 54, 56, 57, 60, 61, 62, 63, 65, 69, 70, 71, 72, 77, 81, 82, 86, 87, 88, 91, 93, 95, 96, 97, 99, 100, 101, 103, 104, 106, 107, 108, 109, 110, 111, 112, 113, 114, 115, 116, 117, 118, 119, 120, 121, 122, 123, 124, 125, 126, 127, 128, 129, 130, 131, 132, 133, 134, 135, 136, 137, 138, 139, 141, 145, 146, 148, 150, 151, 152, 154, 156, 157, 159, 164, 165, 166, 167, 168, 169, 172, 173, 174] were published between 2017 and 2025 following the availability of generic PrEP [175, 176].

The time horizons of the included studies ranged from 3 months to lifetime. Approximately a quarter (*n* = 29; 23%) of included studies used a lifetime time horizon [48, 55, 59, 71, 76, 77, 82, 83, 84, 85, 86, 88, 101, 109, 111, 114, 115, 125, 134, 137, 145, 146, 147, 149, 151, 152, 154, 161, 169]. Fourteen studies employed time horizons of 5 years or shorter [58, 65, 93, 98, 99, 100, 103, 128, 129, 144, 148, 155, 162, 174]. Ninety‐four studies (73%) [7, 51, 52, 53, 54, 57, 59, 62, 64, 65, 67, 69, 70, 72, 73, 75, 76, 77, 78, 79, 80, 81, 82, 83, 87, 90, 91, 92, 93, 94, 96, 97, 98, 102, 103, 104, 105, 106, 107, 108, 109, 110, 111, 112, 113, 114, 115, 117, 119, 120, 121, 123, 124, 125, 126, 127, 128, 129, 130, 131, 132, 133, 134, 136, 137, 138, 139, 141, 142, 143, 144, 146, 147, 148, 150, 151, 152, 153, 156, 157, 158, 159, 160, 164, 165, 166, 167, 168, 169, 170, 171, 172, 173, 174] simulated HIV epidemics that included a time‐period in 2015 or later, with 65 studies [51, 52, 54, 59, 67, 69, 70, 72, 75, 76, 77, 79, 80, 81, 82, 83, 91, 92, 96, 102, 104, 106, 107, 108, 109, 110, 111, 112, 113, 114, 115, 117, 119, 120, 121, 123, 124, 125, 126, 127, 130, 131, 132, 133, 134, 136, 139, 141, 146, 147, 150, 151, 152, 153, 156, 157, 159, 160, 165, 166, 167, 170, 171, 172, 173] simulating epidemics and outcomes in 2030 or later (up to 2103 [115]). One study simulated an HIV epidemic prior to 2015 [58]. The remaining 33 (26%) studies did not report the year(s) of HIV epidemic captured in the model [48, 49, 50, 55, 56, 60, 61, 63, 66, 68, 71, 74, 84, 85, 86, 88, 89, 95, 99, 100, 101, 116, 118, 122, 135, 140, 145, 149, 154, 155, 161, 162, 163].

### 3.4 Distribution of PrEP economic evaluations by perspective of analysis and year of publication

**Figure 2:**
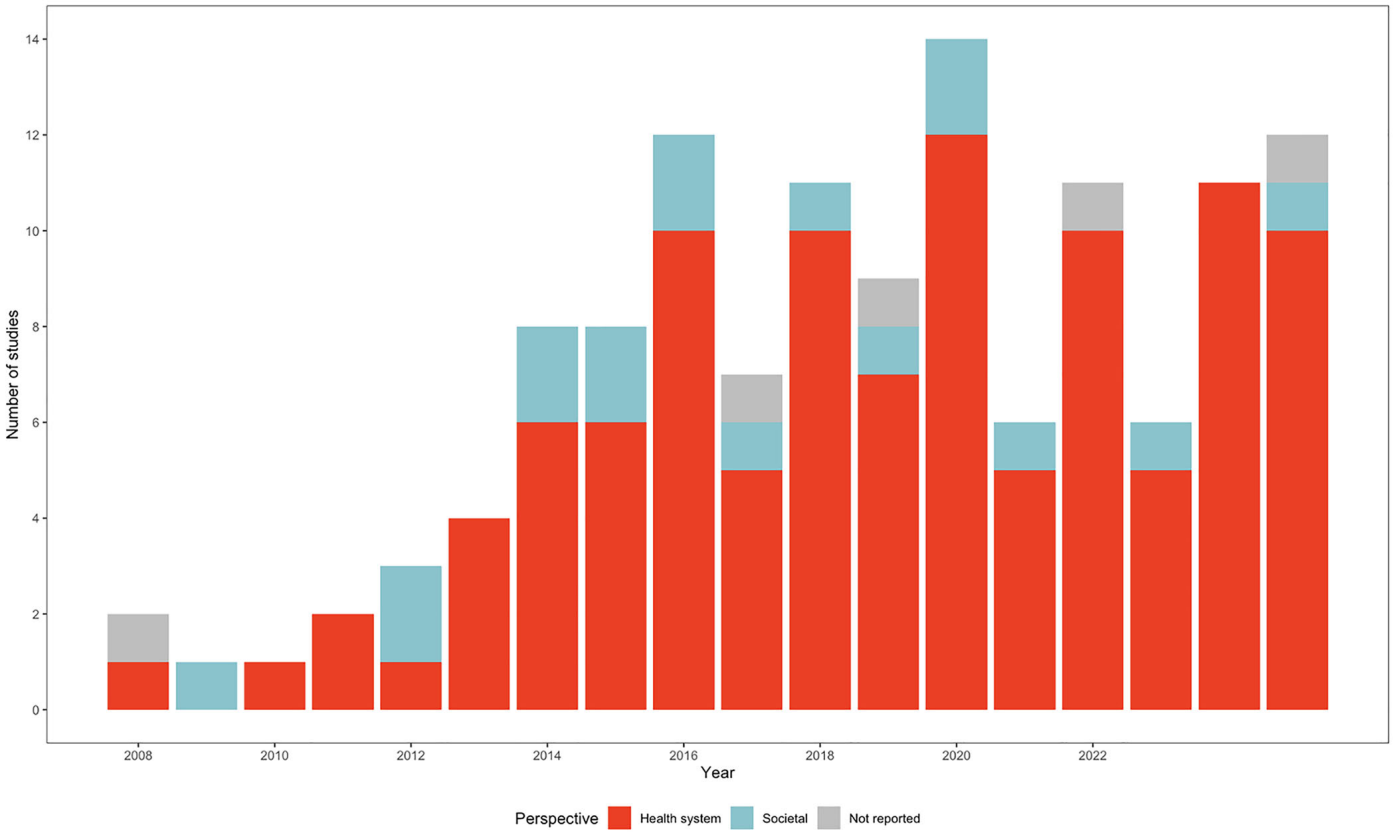
Number of PrEP cost‐effectiveness studies by perspective of the analysis and year of publication (*N* = 128). Abbreviation: *N*, number of studies.

One hundred and six of the 128 (83%) included studies conducted analyses from a health system perspective [7, 48, 49, 51, 53, 54, 56, 57, 58, 60, 62, 63, 64, 66, 68, 69, 70, 71, 72, 73, 74, 75, 76, 77, 78, 79, 80, 81, 82, 85, 86, 87, 89, 90, 91, 92, 93, 94, 96, 97, 98, 99, 100, 102, 103, 105, 108, 109, 111, 112, 113, 114, 115, 116, 117, 118, 119, 120, 121, 122, 123, 124, 125, 126, 127, 128, 129, 131, 132, 134, 135, 136, 137, 138, 139, 140, 141, 142, 143, 144, 145, 146, 148, 149, 150, 151, 152, 153, 154, 156, 157, 158, 159, 160, 162, 164, 165, 166, 167, 168, 169, 170, 171, 172, 173, 174]. These studies captured healthcare costs and associated health outcomes within the context of the health system (e.g. costs covered by a public healthcare payer). From 2013 to 2025 (i.e. following the first authorized use of PrEP by the US FDA), 71−100% of PrEP cost‐effectiveness studies each year were conducted from a health system perspective (Figure 2).

Less than one fifth of included studies conducted analyses from a societal perspective (*n* = 17, *n* = 13%) and examined the full range of costs and outcomes associated with PrEP use including productivity costs [50, 52, 55, 59, 61, 65, 67, 83, 84, 88, 101, 104, 110, 133, 147, 155, 161]. Five studies did not report the perspective taken for analysis [95, 106, 107, 130, 163].

### 3.5 Economic evaluation of PrEP by region, population who received PrEP and populations included in outcome measures

**Figure 3:**
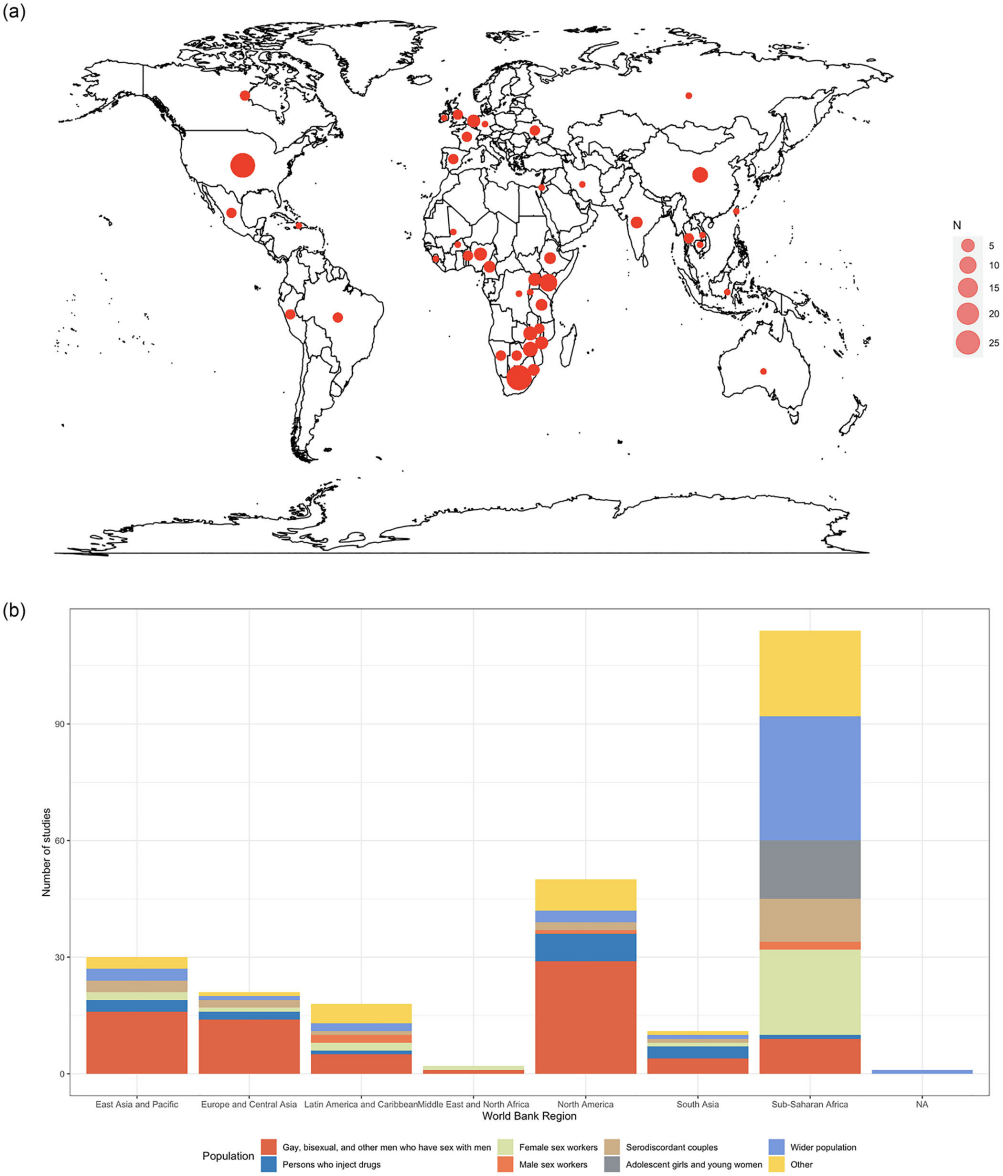
(a) Geographic location of PrEP intervention and implementation strategy cost‐effectiveness models (*N* = 128). Abbreviation: *N*, number of studies. *Note*: Six studies were conducted in the sub‐Saharan African region, but did not specify the countries assessed, so they are not shown on the map. (b) Number of studies by World Bank region and study population who received PrEP (*N* = 128). “Other” includes: those with early HIV detection; transgender women; partners of persons who inject drugs; White and Black adolescent sexual minority males; adolescents and young adults; clients of female sex workers; and female partners of miners. Abbreviations: *N*, number of studies; NA, not applicable. *Note*: Some studies involved multiple populations, so the total number of studies adds up to more than 128.

Fifty‐one (40%) studies modelled HIV epidemics in countries across sub‐Saharan Africa (Figure 3a) [49, 51, 57, 66, 73, 75, 76, 78, 79, 85, 86, 92, 94, 98, 99, 102, 105, 113, 117, 119, 126, 127, 130, 131, 136, 138, 139, 141, 142, 143, 144, 146, 147, 148, 149, 150, 156, 157, 158, 159, 160, 161, 162, 165, 167, 169, 170, 171, 172, 173, 174]. Approximately a quarter of the 128 included studies modelled HIV epidemics in North America (*n* = 34, 27%) [7, 48, 52, 55, 58, 59, 60, 62, 63, 67, 68, 69, 70, 71, 74, 81, 83, 84, 89, 91, 95, 100, 101, 108, 109, 112, 114, 122, 125, 129, 140, 153, 164, 168]. The remaining studies modelled HIV epidemics in Europe and Central Asia (*n* = 16, 13%) [50, 54, 77, 80, 82, 87, 88, 92, 96, 97, 110, 115, 132, 134, 145, 155], East Asia and Pacific (*n* = 19, 15%) [56, 65, 72, 90, 92, 93, 103, 104, 106, 107, 111, 116, 120, 121, 123, 124, 133, 137, 166], Latin America and Caribbean (*n* = 6, 5%) [53, 64, 92, 118, 128, 154], South Asia (*n* = 4, 3%) [92, 107, 151, 152], and Middle East and North Africa (*n* = 2, 2%) [61, 135].

Except in the context of HIV epidemics across sub‐Saharan Africa, most studies modelled the receipt and use of PrEP among gay, bisexual and other men who have sex with men across HIV epidemics across each of the World Bank regions: 29 (85%) in North America [7, 48, 55, 58, 59, 62, 63, 67, 68, 69, 70, 74, 81, 83, 84, 89, 91, 100, 108, 109, 112, 114, 122, 125, 129, 140, 153, 164, 168], 14 (88%) in Europe and Central Asia [54, 77, 80, 82, 87, 88, 92, 96, 97, 110, 115, 132, 134, 145], 16 (84%) in east Asia and Pacific [56, 65, 72, 90, 92, 93, 103, 106, 107, 116, 120, 121, 123, 133, 137, 166], four (67%) in Latin America and Caribbean [64, 92, 118, 154], four (100%) in South Asia [92, 151, 152] and one (50%) in Middle East and North Africa [61] (Figure 3b).

In contrast, two‐thirds (*n* = 34, 67%) of the studies modelling HIV epidemics in sub‐Saharan African countries examined PrEP use in the wider population, defined as the total adult male and/or female population in a region (Figure 3b) [49, 51, 73, 79, 85, 86, 92, 94, 98, 102, 113, 117, 127, 130, 131, 139, 141, 143, 146, 148, 150, 156, 158, 159, 160, 161, 162, 165, 167, 170, 171, 172, 173, 174]. In studies modelling HIV epidemics across sub‐Saharan Africa, PrEP receipt and use was also assessed in the following populations: female sex workers (*n* = 23, 45%) [57, 86, 92, 113, 117, 119, 126, 127, 130, 138, 141, 147, 148, 150, 156, 157, 159, 160, 165, 167, 170, 171, 173], male sex workers (*n* = 2, 4%) [57, 117], HIV‐serodifferent partners (*n* = 12, 24%) [66, 75, 78, 92, 105, 127, 131, 142, 144, 147, 149, 157], adolescent girls and young women (*n* = 15, 29%) [51, 86, 117, 127, 131, 141, 148, 156, 157, 159, 161, 162, 167, 169, 173], gay, bisexual and other men who have sex with men (*n* = 10, 20%) [57, 92, 94, 119, 136, 150, 165, 167, 170, 171] and persons who inject drugs (*n* = 2, 4%) [92, 165].

Across the 128 included studies, particularly studies modelling World Bank regions other than sub‐Saharan Africa, few studies assessed the cost‐effectiveness of PrEP among other key populations including HIV‐serodifferent partners (*n* = 15, 12%) [66, 71, 75, 78, 92, 104, 105, 124, 127, 131, 142, 144, 147, 149, 153, 155, 157], persons who inject drugs (*n* = 12, 9%) [50, 52, 60, 69, 70, 92, 95, 112, 151, 152, 153, 165], female sex workers (*n* = 26, 20%) [57, 86, 92, 113, 117, 118, 119, 126, 127, 130, 135, 138, 141, 147, 148, 150, 156, 157, 159, 160, 165, 166, 167, 170, 171, 173], male sex workers (*n* = 6, 3%) [57, 63, 64, 117, 118, 128], and adolescent girls and young women (*n* = 15, 12%) [51, 86, 117, 127, 131, 141, 148, 156, 157, 159, 161, 162, 167, 169, 173].

Overall, 102 studies (80%) [7, 49, 50, 51, 52, 53, 54, 56, 57, 58, 59, 60, 61, 62, 63, 64, 65, 66, 67, 68, 69, 70, 71, 72, 73, 74, 75, 76, 78, 79, 80, 81, 84, 85, 87, 89, 90, 91, 92, 93, 94, 96, 97, 98, 101, 102, 103, 104, 105, 106, 108, 109, 110, 111, 112, 113, 114, 115, 117, 119, 120, 122, 125, 126, 127, 130, 131, 132, 133, 134, 136, 138, 139, 140, 141, 142, 143, 144, 146, 147, 148, 149, 150, 151, 152, 153, 154, 155, 156, 157, 158, 159, 160, 161, 162, 163, 165, 167, 169, 170, 171, 173] included transmission dynamics (i.e. indirect benefit to partners of people who received PrEP) and 26 (20%) [48, 55, 77, 82, 83, 86, 88, 95, 99, 100, 107, 116, 118, 121, 123, 124, 128, 129, 135, 137, 145, 164, 166, 168, 172, 174] did not.

### 3.6 Economic evaluations by PrEP modality

Most studies (*n* = 99, 77%) assessed oral PrEP, of which 76 (77%) assessed daily oral PrEP [7, 48, 49, 50, 52, 55, 56, 58, 59, 61, 62, 64, 65, 66, 67, 71, 72, 74, 76, 77, 78, 79, 80, 82, 85, 86, 87, 88, 89, 90, 93, 96, 97, 98, 100, 101, 103, 104, 106, 107, 109, 110, 111, 113, 114, 115, 116, 117, 120, 121, 122, 123, 124, 125, 126, 127, 128, 129, 132, 133, 134, 136, 144, 148, 150, 151, 153, 154, 156, 158, 159, 160, 162, 165, 167, 173], 16 (16%) assessed on‐demand oral PrEP [54, 61, 80, 83, 88, 89, 97, 106, 107, 121, 122, 129, 133, 134, 136, 145] and 20 (20%) assessed oral PrEP without specifying daily oral or on‐demand [51, 53, 57, 60, 63, 70, 73, 84, 91, 99, 131, 140, 149, 157, 161, 166, 170, 171, 172, 174]. Seventeen (13%) of the 128 included studies evaluated long‐acting injectable PrEP [86, 99, 109, 113, 121, 122, 126, 129, 130, 132, 137, 139, 147, 162, 167, 169, 173]. Seven (5%) studies evaluated other PrEP modalities (e.g. vaginal ring, topical gel) [86, 94, 102, 146, 159, 161, 163]. Twenty (16%) studies did not specify the PrEP modality (i.e. oral, injectable, vaginal ring, topical gel) evaluated [68, 69, 75, 81, 92, 95, 105, 108, 112, 118, 119, 135, 138, 141, 142, 143, 152, 155, 164, 168].

### 3.7 Type of comparators used over time in economic evaluations of PrEP

**Figure 4:**
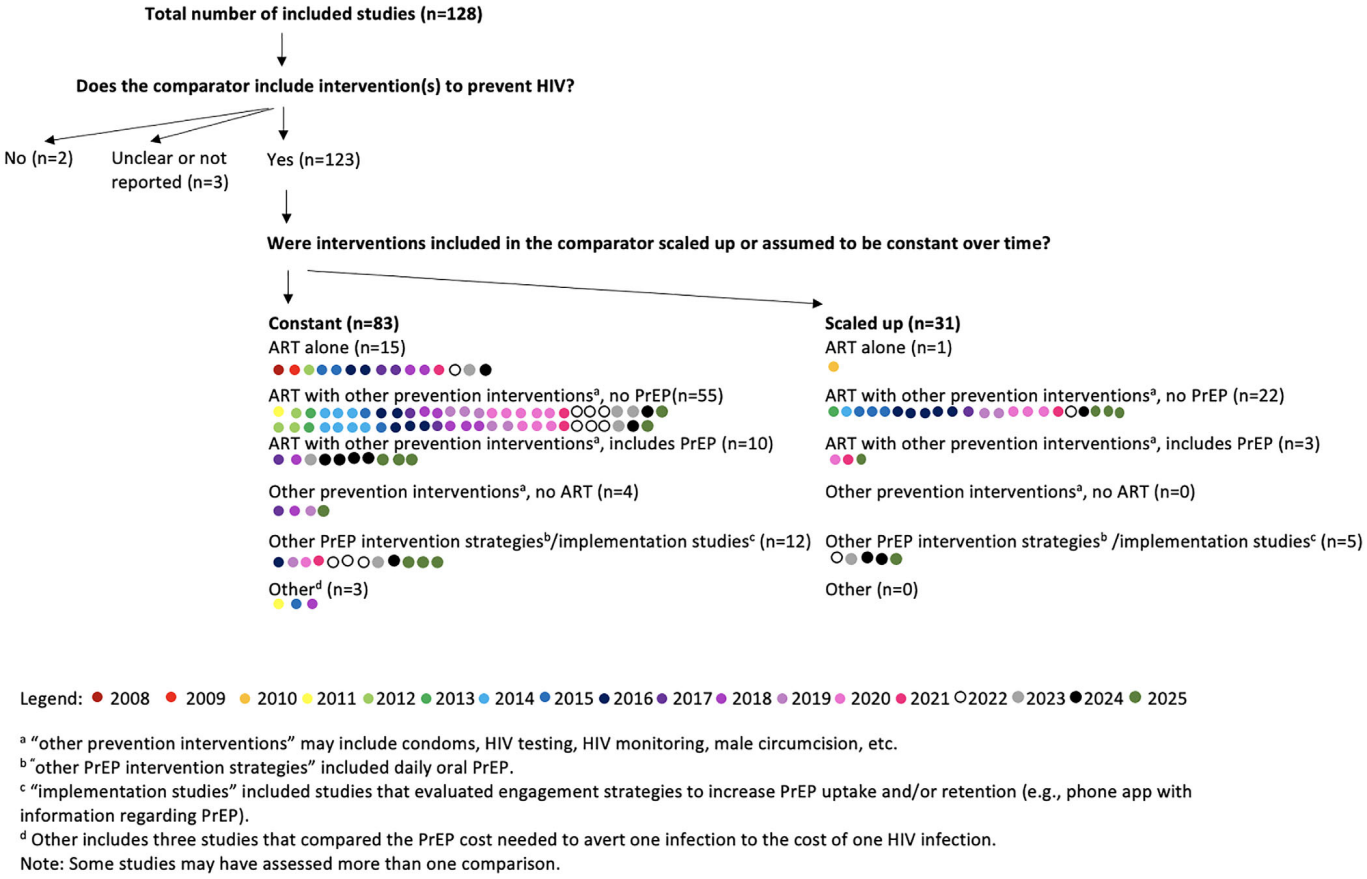
Types of comparators among included studies by applicability to real‐world practice and year of study publication.

Of the 123 studies that included at least one HIV prevention intervention in the comparator, the most common comparator was a combination of ART and other HIV prevention interventions excluding PrEP (*n* = 77; 63%; Figure 4) [49, 50, 51, 53, 54, 55, 56, 57, 59, 60, 61, 62, 63, 64, 66, 67, 69, 72, 74, 76, 78, 79, 80, 87, 90, 92, 93, 94, 95, 96, 97, 98, 99, 101, 104, 105, 106, 109, 110, 111, 114, 116, 117, 118, 122, 123, 127, 131, 132, 133, 136, 137, 139, 140, 142, 143, 144, 146, 147, 148, 149, 150, 151, 152, 153, 154, 155, 156, 157, 160, 161, 162, 165, 170, 171, 172, 174]. Sixteen studies (13%), published between 2008 and 2024, only included ART in the comparator (i.e. no other concomitant HIV prevention interventions) [58, 64, 68, 71, 75, 77, 82, 84, 85, 89, 103, 119, 120, 124, 141, 158]. Four (3%) studies published from 2017 to 2025 did not include ART but included other concomitant HIV prevention interventions (e.g. condoms, HIV testing, HIV counselling, HIV monitoring, harm reduction and/or male circumcision) in their comparator [48, 65, 86, 135]. One of the studies simulated an HIV epidemic between 2009 and 2019 [65], while the other three studies did not report the time period of the HIV epidemics simulated [48, 86, 135]. Studies (*n* = 13, 11%) that included PrEP along with other HIV prevention interventions in the comparator were all published in 2017 or later [52, 81, 91, 112, 115, 121, 126, 130, 134, 138, 164, 166, 169].

Assumptions regarding background HIV incidence varied, with 49 studies modelling declining incidence [48, 49, 50, 51, 53, 54, 56, 57, 61, 64, 65, 67, 72, 73, 78, 79, 80, 81, 82, 87, 97, 105, 109, 113, 115, 117, 119, 120, 126, 131, 133, 134, 136, 138, 139, 142, 143, 144, 147, 150, 151, 152, 157, 158, 160, 167, 168, 170, 172], 32 modelling stable incidence [55, 58, 74, 77, 81, 85, 86, 89, 90, 95, 101, 102, 106, 111, 114, 116, 121, 122, 124, 128, 129, 132, 149, 154, 156, 161, 162, 163, 164, 165, 169, 173] and 10 modelling increasing incidence [59, 62, 84, 91, 94, 96, 103, 135, 139, 165, 174]. Despite modelled changes in background HIV incidence, 55 of the 77 studies (71%) that compared PrEP to ART and other HIV prevention interventions excluding PrEP assumed constant use rates over time [49, 50, 51, 53, 54, 55, 56, 59, 60, 61, 62, 63, 64, 66, 67, 72, 74, 76, 78, 79, 80, 90, 93, 94, 95, 96, 97, 98, 99, 101, 106, 109, 110, 111, 114, 116, 117, 118, 122, 123, 127, 132, 133, 148, 149, 150, 151, 152, 153, 154, 155, 156, 161, 162, 165]. Of the 123 studies that included at least one HIV prevention intervention in the comparator, 26 (21%) considered the scale‐up (i.e. increased coverage and increased uptake) of ART [57, 69, 81, 87, 92, 104, 105, 112, 113, 130, 131, 136, 139, 140, 142, 143, 144, 146, 147, 157, 158, 160, 170, 171, 172, 174], and 20 studies (16%) considered increased coverage and/or uptake of other prevention interventions over time [57, 81, 92, 107, 112, 130, 131, 137, 140, 142, 143, 144, 146, 147, 157, 160, 170, 171, 172, 173].

### 3.8 Economic evaluations comparing PrEP modalities and implementation strategies

Twelve studies (9%) evaluated the cost‐effectiveness of one PrEP modality versus another PrEP modality (Figure 4) including: (1) nine studies that compared long‐acting injectable PrEP to daily or on‐demand oral PrEP and other concomitant interventions (published between 2016 and 2025) [109, 113, 122, 129, 130, 159, 162, 167, 173]; (2) a study that compared dapivirine vaginal rings to daily oral PrEP with concomitant ART, condom use and voluntary male circumcision (published in 2019) [159]; and (3) two studies that compared branded to generic daily oral PrEP without any concomitant interventions in the comparator (published in 2020) [100, 107].

Only five studies (4%), published between 2021 and 2025, examined the cost‐effectiveness of different PrEP implementation strategies [7, 108, 125, 128, 168]. The five studies evaluated the cost‐effectiveness of PrEP with and without PrEP engagement interventions (i.e. interventions to improve PrEP initiation, adherence and persistence). PrEP initiation interventions assessed included: (1) a phone app with information about PrEP, including individualized risk assessments and PrEP provider locations [7]; and (2) a phone app that assessed PrEP eligibility among gay, bisexual and other men who have sex with men, and allowed users to order HIV/STI tests and safe sex products [125]. PrEP adherence interventions assessed included: (1) counselling [7, 125]; (2) text message support [108]; (3) PrEP patient navigation via trained healthcare professionals and navigators; (4) educational training and PrEP screening questionnaires to help physicians integrate PrEP consultation and referral into visits [108]; and (5) economic incentives in the form of supermarket vouchers [128]. Two studies evaluated the use of mobile phone apps that sought to facilitate telemedicine visits with PrEP providers to improve PrEP persistence [7, 125]. One study tested direct‐to‐consumer via a digital HIV prevention intervention versus community‐based organization to improve engagement (initiation, adherence and persistence) in HIV preventions, including PrEP [168].

## 4 DISCUSSION

Our review identified 128 economic evaluations of PrEP, with a rapidly growing number of studies over time. It highlights key areas for future work in assessing the cost‐effectiveness of PrEP policy, programmes and person‐centred decisions. Almost three‐quarters of published economic evaluations of PrEP examined HIV epidemics in 2015 or later, following WHO endorsement of PrEP and the increasing number of countries that have authorized the use of PrEP [9]. We identified a paucity of studies in the following areas: quantifying value for money from a broader societal perspective; capturing diverse epidemic context (geographic regions and populations); and evaluating different PrEP modalities and implementation strategies. Finally, our review found that only 31 of 128 included at least one HIV prevention intervention in the comparator and accounted for the concurrent scale‐up of other interventions, highlighting an area for future scenario analyses where comparators may also change over time.

Emerging PrEP modalities, particularly long‐acting injectable regimens, introduce costs (healthcare, transportation, accommodation, caregiving, productivity loss) and benefits (PrEP uptake and adherence, prevention of HIV transmission) that differ from existing or no PrEP scenarios [167]. These differences underscore the importance of expanding the time horizon and perspective of economic evaluations to include societal impacts to capture both immediate and downstream health benefits and quality of life, as well as broader societal gains such as improved productivity and the spillover effects of improved public health [177, 178]. Findings from included cost‐effectiveness modelling studies highlight that, while the introduction of PrEP may increase costs in the short and medium term, the implementation of PrEP becomes increasingly cost‐effective—and eventually cost‐saving—when evaluated over longer time horizons [109, 116]. These findings reflect a well‐established phenomenon where the value for money of prevention measures accrue over time, and thus, underscore the importance of adopting sufficiently long analytic periods in economic evaluations of PrEP strategies [54, 109]. Previous costing studies have shown that PrEP can enhance work productivity by reducing the risk of HIV transmission [177, 179, 180]. Economic evaluations that only consider health system perspectives may underestimate PrEP's value for money [181], potentially leading to suboptimal resource allocation decisions. As future economic analyses of PrEP modalities and/or PrEP implementation are developed, adopting a societal perspective would align more directly with the current era of an expansive choice in PrEP modalities, thereby supporting decision‐making surrounding “person‐centred” HIV prevention [182].

Our findings suggest important areas for future economic evaluations to keep pace with the evolving HIV response. Chief among them is the examination of the cost‐effectiveness of different implementation strategies and PrEP modalities—particularly newer modalities such as long‐acting injectable PrEP [183, 184]. Economic evaluations are often used to inform clinical guidelines and resource allocation in a landscape of shrinking resources. The limited evaluation of newer PrEP modalities likely reflects timing: new modalities were recently approved in various jurisdictions and are not yet widely available in many settings, particularly in low‐ and middle‐income countries [185]. Programmatic and policy decisions have shifted from the “technology” (i.e. whether PrEP should be offered), particularly among populations experiencing disproportionate risk, to implementation in an era of choice, with a focus on long‐acting injectable PrEP. Implementation includes focusing on how to reach those who would benefit at an individual‐level or those who may be part of sexual and/or injecting networks so that PrEP use can reduce population‐level HIV transmission, as well as how to support user choices across regimens (building upon previous binary comparisons of oral PrEP vs. long‐acting injectable PrEP) [167]. Despite the adoption of PrEP in several national and international guidelines [10, 11], PrEP coverage [12, 186], uptake [186] and adherence [187] remain programmatic challenges in many regions. In 2020, PrEP coverage was estimated at 8% of the estimated global need, and 28% of the estimated need in low‐ and middle‐income countries [186]; with inequities in PrEP coverage by race, age, geography and socio‐economic status [188]. As empirical data on the comparative reach and effectiveness of implementation strategies emerge, economic evaluations of implementation strategies delivering on choice can support the selection and scale‐up of effective and efficient programmes. These include, but are not limited to, estimating the maximum pricing of PrEP and the pricing of programme/service delivery, for PrEP programmes to be considered good value for money by a country's health system [173].

Alongside the expansion of PrEP modalities is the integration of HIV prevention services and programmes within a broader mandate of universal healthcare and service provision. This is taking shape alongside an evolving epidemic response and epidemic dynamics surrounding populations experiencing disproportionate risks of HIV acquisition [189]. Across many priority populations, HIV prevention programmes are part of community‐based and community‐led programmes [190]. In many countries, particularly in low‐ and middle‐income countries, population‐focused HIV prevention programmes may be increasingly integrated within the larger health sector, such that PrEP implementation strategies may change over time, along with cost and effectiveness of such strategies [191]. These dynamics were already in place and are even more pertinent now in low‐ and middle‐income countries with disruptions and adaptations of HIV prevention and care services with large‐scale funding disruptions in donor support from high‐income countries like the United States [192, 193, 194]. Thus, decisions are underway on how to prioritize HIV prevention, especially PrEP implementation and modalities, given changes anticipated across comparators over time. Thus, in anticipation of uncertainties in the future coverage of HIV prevention and treatment programmes, future analyses would benefit from considering the potential scale‐up or decline in comparator interventions to support decision‐making. These scenarios may become increasingly relevant in the era of integration of HIV prevention and treatment programmes with the wider health system, given how influentialthe time‐horizon is in estimates of cost‐effectiveness of prevention interventions [54, 109, 116, 177, 178].

While our review found that a range of regions and populations have been considered in PrEP economic evaluations, most focused on countries in sub‐Saharan Africa and a range of underlying HIV risks among gay, bisexual and other men who have sex with men. There was a paucity of studies focused on Latin America and the Caribbean, as well as South Asia [195]. Additionally, there remain subsets of the population at disproportionate HIV risk among whom future work could be conducted to support decision‐making given heterogeneity in HIV risks and service delivery (programme implementation): including but not limited to individuals engaged in sex work (formal and informal); men who pay for sex (clients of sex workers); people who inject drugs; adolescents and young women; and transgender women [196, 197]. Ensuring PrEP (across its modalities) is accessible to and reaches those who could benefit also means considering health equity as one of the population‐level goals (in addition to a reduction in population‐level transmission). Such goals would benefit from integrating efforts at examining distributional cost‐effectiveness and equity‐informed economic evaluations [198]. Taken together, there remains an important opportunity for future economic evaluations to build on the prior literature, with a focus on likely programmatic shifts, current prevention gap, and comparators that mirror the current and potential trajectory (scale‐up) of concomitant combination HIV prevention coverage over time.

### 4.1 Limitations

Our study had several limitations. First, our review was restricted to peer‐reviewed publications and did not include grey literature, such as reports from health technology assessment agencies, which often inform health system decisions. As such, our study may not fully represent the breadth of economic evaluations conducted, potentially overlooking some data used in policy decision‐making. Second, the lack of detailed reporting in some studies hindered our ability to extract all variables of interest, resulting in gaps in our synthesis. Although earlier forms of methodological guidance for economic evaluations existed [199], many studies included in our review predated the introduction and broader adoption of standardized reporting guidelines, which have since improved transparency and consistency in reporting [200, 201]. Studies that predated the adoption of reporting guidelines often omitted several key elements necessary for assessing methodological quality. This deficiency raises concerns about the reliability of their findings. Future work, as proposed, includes conducting a more detailed methodological review to assess the validity and reliability of included studies [44].

## 5 CONCLUSIONS

Economic evaluations of HIV PrEP have grown over time. There remain important areas for future economic evaluations to prioritize, including consideration of societal costs and benefits, the broader impacts of PrEP, choice across a growing expanse of PrEP modalities, comparison across PrEP implementation strategies and the most relevant comparators as the HIV epidemic dynamics evolve. The global HIV response is at a critical juncture, with integration into the universal healthcare mandate, overall reduction in funding and resources, sustainability and acceleration to end HIV as a public health threat, and a re‐centring of health equity given persistent disproportionate risks among populations experiencing social, economic and structural marginalization [189, 202]. Programmatic and policy decisions that use estimates of value for money will need economic evaluations that mirror the current state of HIV prevention implementation and HIV epidemic realities, and those anticipated in the near future.

## COMPETING INTERESTS

DHST's institution has received support from Gilead for investigator‐initiated research and from GlaxoSmithKline for participation in industry‐sponsored clinical trials. No other authors have any conflicts of interest.

## AUTHOR CONTRIBUTIONS

DHST, SDB, HK, BS, DRM, KT and SM conceived the review. BS, KT and SM developed the search strategy. BS conducted the literature search. MX, KT and SM screened titles and abstracts. MX, HK, KT and SM screened full texts. MX, HK, KT and SM developed the data extraction protocol and designed the data extraction spreadsheet. MX, LM and HK extracted the data. MX synthesized the data and drafted the manuscript. All authors have read and approved the final manuscript.

## FUNDING

This study was funded by a Ontario Ministry of Colleges, Universities, Research Excellence and Security Early Researcher Award (ER17‐13‐043), a Canadian Institutes for Health Research Foundations Award (FDN‐143266) and a Canadian Institutes for Health Research Project Grant (416186).

## Supporting information



Appendix A. PRISMA checklist
Appendix B. Search strategy

Appendix C. Data extraction form.

## Data Availability

All data used in this systematic review are derived from previously published studies, which are cited in the manuscript. Extracted data are available in our Supplementary Material.
